# NIRS-ICA: A MATLAB Toolbox for Independent Component Analysis Applied in fNIRS Studies

**DOI:** 10.3389/fninf.2021.683735

**Published:** 2021-07-14

**Authors:** Yang Zhao, Pei-Pei Sun, Fu-Lun Tan, Xin Hou, Chao-Zhe Zhu

**Affiliations:** ^1^State Key Laboratory of Cognitive Neuroscience and Learning, Beijing Normal University, Beijing, China; ^2^IDG/McGovern Institute for Brain Research, Beijing Normal University, Beijing, China

**Keywords:** functional near-infrared spectroscopy, data processing, multivariate analysis, independent component analysis, blind source separation, MATLAB toolbox, software

## Abstract

Independent component analysis (ICA) is a multivariate approach that has been widely used in analyzing brain imaging data. In the field of functional near-infrared spectroscopy (fNIRS), its promising effectiveness has been shown in both removing noise and extracting neuronal activity-related sources. The application of ICA remains challenging due to its complexity in usage, and an easy-to-use toolbox dedicated to ICA processing is still lacking in the fNIRS community. In this study, we propose NIRS-ICA, an open-source MATLAB toolbox to ease the difficulty of ICA application for fNIRS studies. NIRS-ICA incorporates commonly used ICA algorithms for source separation, user-friendly GUI, and quantitative evaluation metrics assisting source selection, which facilitate both removing noise and extracting neuronal activity-related sources. The options used in the processing can also be reported easily, which promotes using ICA in a more reproducible way. The proposed toolbox is validated and demonstrated based on both simulative and real fNIRS datasets. We expect the release of the toolbox will extent the application for ICA in the fNIRS community.

## Introduction

Functional near-infrared spectroscopy (fNIRS) is a non-invasive optical imaging technology that has been widely used in studying human brain activity (Boas et al., 2014). It has been utilized to investigate the task-evoked neuronal response of various cognitive functions as well as spontaneous neural activity, which is reflected by resting-state functional connectivity (White et al., 2009). Compared with fMRI, fNIRS is portable, less sensitive to head motion, and has high ecological validity, which make it very suitable to study human social interactions by hyper-scanning multiple brains in naturalistic environment, and special populations such as infants, patients, and the elderly population (Ferrari and Quaresima, 2012; Scholkmann et al., 2013). It also has higher temporal resolution and a more comprehensive measurement for hemoglobin changes in the cerebral cortex, which potentially provides a deeper understanding of the neurovascular coupling process (Steinbrink et al., 2006).

However, besides the sources of neural activity, various physiological and non-physiological noise components have been found in fNIRS signals (Scholkmann et al., 2014). Since fNIRS detects brain activity based on the banana-shaped light path traveled through both extra- and intra-cerebral compartments, the neuronal activity-unrelated physiological changes, such as heart rate, respiration, Mayer waves, and blood pressure, in these compartments can induce changes in optical intensity, and further included as noise components in fNIRS signals (Liebert et al., 2004). Moreover, although fNIRS is less sensitive to head motion than fMRI, motion artifacts, generated from optode decoupling with the scalp, are still often presented in many fNIRS studies (Robertson and Douglas, 2010; Brigadoi et al., 2014; Cui et al., 2015). These latent noise processes are inherent in fNIRS signals and strongly reduce the quality of the recorded data. Therefore, the reduction of noises and extraction of neuronal activity-related components are essential for the fNIRS studies.

The methodology for processing fNIRS data is divided into two classes, i.e., univariate and multivariate methods by Scholkmann et al. (2014). Univariate methods independently process the data of each channel. These approaches include pre-processing methods such as bandpass or wavelet filtering and analysis methods like general linear model (GLM) regression, which have been implemented in public-available software such as NIRS-SPM and Homer2 (Huppert et al., 2009; Ye et al., 2009). Multivariate methods, another type of analysis method, have also been proposed and applied for fNIRS. Instead of processing data of each fNIRS channel separately, multivariate methods additionally exploit the correlation between the data of different channels and have shown better performance in removing noise or extracting neuronal activity-related sources compared to univariate methods (Scholkmann et al., 2014).

Independent component analysis (ICA) is a multivariate approach for processing fNIRS data. Two ways of using ICA had been presented in fNIRS literature. First, as a preprocessing method, it has been used to remove noises including both motion artifacts and physiological noises (Kohno et al., 2007; Robertson and Douglas, 2010; Virtanen et al., 2011; Chaddad, 2014). Second, ICA has been applied as a data-driven analysis method for exploring the neuronal activity-related sources, or sources of interest (SOI), including event-related fast optical signal, task-evoked hemodynamic response, and resting-state functional connectivity in typical functional systems (Morren et al., 2004; Katura et al., 2008; Medvedev et al., 2008; Markham et al., 2009; Zhang et al., 2010). For task data analysis, it can extract task-evoked hemodynamic responses without using a prior-defined hemodynamic response function (HRF), as in the conventional regression-based method, i.e., GLM. For analyzing resting-state functional connectivity, compared with the seed-based correlation analysis, ICA does not have the bias that originated from the selection of seed channels. Since the noises have been separated while extracting the SOI, the ICA approach usually outperforms these traditional methods (Kohno et al., 2007; Markham et al., 2009; Zhang et al., 2010).

Some of the fNIRS software packages have already included ICA as a module of their analysis pipeline (Strangman et al., 2009; Raggam, 2020). However, the usages of ICA in these toolboxes are limited to certain types of fNIRS devices, or processing steps (e.g., preprocessing). Therefore, a toolbox that can fully unveil the potential of ICA analysis in the fNIRS field is still lacking. In this study, we propose NIRS-ICA, a user-friendly, systemic integrated, public-available MATLAB toolbox to ease the ICA application for the fNIRS dataset. NIRS-ICA integrates commonly used ICA algorithms for source separation and provides user-friendly GUIs which can be used for both noise reduction and extracting SOI. In addition, NIRS-ICA also incorporates quantitative evaluation metrics to evaluate the separated sources. Therefore, users can speed up the procedure of source selection by ranking the sources based on the value of the metrics. The options used in the processing can also be output as a report easily, which facilitates reproducing the ICA method in another study.

This manuscript is structured as follows: In Section “Materials and Methods,” the mathematical model and applications of ICA used in the NIRS-ICA are first illustrated (sections “Mathematical Model of ICA” and “Applications”). Then we present the simulative and real fNIRS dataset used for validating the functionality of noise reduction and extracting SOI (section “Validations”). In Section “Implementations and Results,” the developed GUIs and usages of NIRS-ICA for noise reduction and extracting SOI are demonstrated based on the simulative and real fNIRS datasets, respectively. The results of the simulative and real fNIRS experiments are also compared with conventional analysis methods. Finally, the proposed approach and toolbox are discussed in Section “Discussion.”

## Materials and Methods

### Mathematical Model of ICA

**FIGURE 1:**
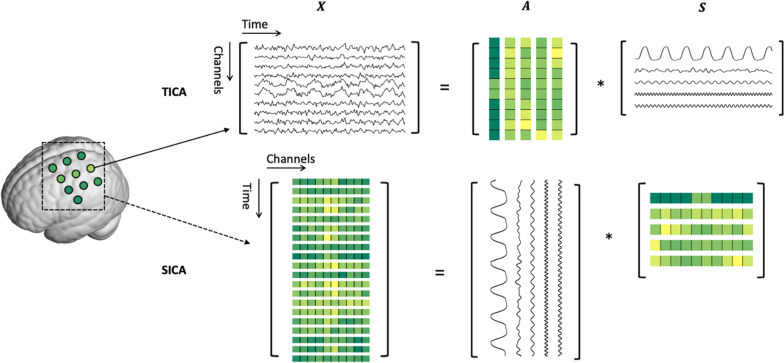
Schematic depiction of temporal and spatial ICA for fNIRS data.

The mathematical model of ICA applied in fNIRS data analysis is briefly introduced as follows: In general, ICA is a data-driven method that decomposes the data of fNIRS into multiple source components and their corresponding weights, which is mathematically expressed as:

(1) X=A⋅S,

where **X** is the matrix containing the observed fNIRS data. **S** and **A** are the matrices of source signals and their associated weights, respectively. Since **A** and **S** are both underdetermined, constraints must be imposed for the decomposition. In the ICA framework, it assumes the source signals in **S** are statistically independent of each other. This constraint can be imposed either on the temporal or spatial dimension of the fNIRS data of a subject, namely, temporal ICA (TICA) or spatial ICA (SICA) (Calhoun et al., 2001b). The bidimensional decomposition model is schematically described in Figure 1. In TICA, each channel of the recorded fNIRS data is considered as a random variable and its values in different time points are regarded as samples. Therefore, each time course of the fNIRS data is modeled as a weighted linear combination of temporally independent source time courses, i.e.,

(2) $$\left[\begin{array}{c} x_{1}\,\left(t\right)\\ \vdots \\ x_{n}\,\left(t\right) \end{array}\right]=\left[\begin{array}{ccc} a_{11}\; & \cdots \; & a_{1\,m}\\ \vdots \; & \ddots \; & \vdots \\ a_{n\,1}\; & \cdots \; & a_{n\,m} \end{array}\right]\cdot \left[\begin{array}{c} s_{1}\,\left(t\right)\\ \vdots \\ s_{m}\,\left(t\right) \end{array}\right],$$

where *x*_*i*_(*t*):(*i*1,2,…,*n*,*t* = 1,2,…,*T*) denotes the observed fNIRS time course of the *i*th channel. *s*_*i*_(*t*):(*i* = 1,2,…,*m*,*t* = 1,2,…,*T*) is the *i*th source time course. The *i*th column of the mixing matrix, i.e., **a**_*i*_ = [*a*_1*i*_,*a*_2*i*_,…,*a*_*ni*_]*T*,contains the contributions of *s*_*i*_(*t*) to the observed signals of *n* fNIRS channels, which represent the spatial map of the source. Note that *m* ≤ *n* otherwise Eq. 2 is underdetermined. In SICA, the fNIRS data of different time points are regarded as different spatial maps, which are weighted linear combinations of spatially independent source spatial maps, i.e.,

(3) $$\left[\begin{array}{c} x_{1}\,\left(s\right)\\ \vdots \\ x_{T}\,\left(s\right) \end{array}\right]=\left[\begin{array}{ccc} a_{11}\; & \cdots \; & a_{1\,m}\\ \vdots \; & \ddots \; & \vdots \\ a_{T\,1}\; & \cdots \; & a_{T\,m} \end{array}\right]\cdot \left[\begin{array}{c} s_{1}\,\left(s\right)\\ \vdots \\ s_{m}\,\left(s\right) \end{array}\right],$$

where *x*_*i*_(*s*):(*i* = 1,2,…,*n*,*s* = 1,2,…,*n*) denotes the spatial map at the *i*th time point. *s*_*i*_(*t*):(*i* = 1,2,…,*m*,*t* = 1,2,…,*T*) is the *i*th source spatial map and *m* ≤ *T*. **a**_*i*_ = [*a*_1*i*_,*a*_2*i*_,…,*a*_*Ti*_]^*T*^ is the time course of the *i*th spatial map. Since the TICA and SICA decompose fNIRS data in different ways, their results should be interpreted correctly. TICA is better for finding latent processes such as motion artifacts and neuronal activity-related hemodynamic responses which are temporally independent which each other, but their spatial distributions can be related. In contrast, SICA identifies sources that happened in independent spatial locations but may have correlated temporal dynamics. Theoretically, both TICA and SICA can be applied to fNIRS data. However, in practice, TICA is more often used than SICA since fNIRS often has few samples in the spatial dimension, i.e., has a small number of channels. Performing ICA with a small number of samples may lead to overfitting problems and result in low reproducibility (Weihong et al., 2012). Therefore, SICA should be used with caution especially when the number of channels is small.

### Applications

#### Decomposition Algorithms

Before ICA, a prewhitening step is performed using principal component analysis (PCA), which means applying a transformation matrix to the data, i.e.,

(4) X=′V⋅X,

where **V** is an orthogonal matrix obtained by singular value decomposition of the covariance matrix of **X** (Huppert et al., 2009). The prewhitening step limits the transformation matrix searched in the ICA step as an orthogonal matrix, which can reduce the computational cost (Hyvarinen et al., 2004). It also reduces the data dimension so that it prevents the over-fitting problem of ICA. The number of sources (*m*) is determined in this step. By default, NIRS-ICA determines the number of sources based on retaining 99% of the data variance using PCA (Zhang et al., 2010). For increasing flexibility, determining the number of sources based on information-theoretic criteria, i.e., Akaike’s information criterion (AIC), Bayesian information criterion (BIC), or users’ input is also implemented. Other preprocessing steps such as detrend and bandpass filtering may also be helpful to achieve an ideal decomposition (Vergara et al., 2017). Therefore, we incorporate commonly used pre-processing methods in the NIRS-ICA package.

Principal component analysis expresses the raw data using orthogonal vectors, which may not be able to fully characterize the true mixing model of sources (McKeown et al., 1998; Calhoun et al., 2009). After the PCA-based prewhitening step, the whitened data are further projected to the sources space for estimation of independent sources, i.e.,

(5) S^=W⋅X,′

where **W** is the unmixing matrix and $\hat{\text{S}}$ is the estimated sources time courses. This procedure can be considered as an optimization problem whose objective function is the statistical independence between sources in $\hat{\text{S}}$. Then the mixing matrix **A** is estimated as $\hat{\text{A}}={\left(\text{W}\cdot \text{V}\right)}^{-1}$. There are mainly two types of ICA algorithms to achieve independence in previous fNIRS studies (Adali et al., 2014). The first type of ICA algorithm involves using high-order statistics (HOS) such as FastICA and Infomax, which decompose **X** by maximizing non-Gaussianity of *s*_*i*_(*t*) or *s*_*i*_(*s*) to achieve independence between source time courses or spatial maps (Hyvarinen, 1999). The second type exploits the sample dependence (SOS). For example, second-order blind identification (SOBI) involves joint diagonalization of the sample-delayed correlation matrix of **X** (Belouchrani et al., 1997). Since FastICA and SOBI are the most commonly used algorithms in fNIRS literature, we incorporate them in the current version of NIRS-ICA. The parameters of the above algorithms are listed in the Supplementary Material. The ambiguity of order, signs, and scales always remain in the sources decomposed by ICA (Hyvarinen et al., 2004). However, only the ambiguity of signs may influence the selection of sources. Therefore, a button to change the sign of a source is implemented in the interface of detailed displaying sources.

#### Source Selection

In the source selection step, the separated sources are manually selected based on their spatial and temporal features. Taking the sources of motion artifacts as an example, the spike-shaped waveforms are often found in their time course, which are generated by the decoupling of the optode and scalp when participants move their heads (Cui et al., 2015). Meanwhile, its corresponding spatial map may present a global pattern since the head motion causes displacement of the optode holder which influences the contact of almost all of the optodes. Other types of noises include heart rate, breath, and Mayer wave, which can generate sources whose time course usually has a narrow frequency spectrum, and spatial map depicts global pattern (Zhang et al., 2010). In NIRS-ICA, we also propose several quantitative metrics to rank the separated sources, which can facilitate the manual source selection procedure. Three metrics are implemented to facilitate identifying noise-related sources. The spike-shaped metric evaluates sources’ time course for identifying motion artifacts based on Scholkmann et al. (2010). Specifically, the moving standard deviation (MSD) is first calculated using the equation:

(6) $$M\,S\,D\,\left(t\right)=\frac{1}{2\,k+1}\,{\left[\sum_{j=-k}^{k}{\hat{s}}^{2}\,\left(t+j\right)-\frac{1}{2\,k+1}\,{\left(\sum_{j=-k}^{k}\hat{s}\,\left(t+j\right)\right)}^{2}\right]}^{\frac{1}{2}},$$

where *t* ∈ {*k* + 1,*k* + 2,…,*T*−*k*} and *T* is the length of the time course. *W* = 2*k* + 1 is the length of the window for calculating the *MSD*(*t*), which can be specified by users. In general, it means that when the time course depicts a high standard deviation, i.e., contains many large spikes, in a time window *W* around time point *t*, it will have high *MSD*(*t*). Therefore, the *W* should be similar to the period of spikes that users aimed to identify. Second, the time points whose *MSD* are outliers, i.e., greater than the threshold: *mean*(*MSD*) ± *n*⋅*std*(*MSD*), are determined as motion artifacts. The default value of *W* and *n* are 2s and 3, respectively. Finally, the value of the spike-shaped metric of one source is derived using the number of outliers identified in its time course.

Correlation with external input (*CEI*) metric evaluates sources by correlating their time courses with the time course recorded by external devices such as accelerometer, physiological instruments, or short channel of fNIRS. Pearson’s correlation coefficient is used for calculating the correlation.

The spatial homogenous metric quantifies the spatial map of sources, which is evaluated based on the coefficient of spatial uniformity (CSU) (Kohno et al., 2007):

(7) $$C\,S\,U\,\left(i\right)=\left|\frac{{\bar{\text{c}}}_{i}}{\sigma \,\left({\text{c}}_{i}\right)}\right|,$$

where ${\bar{\text{c}}}_{i}$ and σ(**c**_*i*_) represent the mean and standard deviation of the spatial map of the *i*th source. Therefore, spatial maps with high mean and low standard deviation will have high CSU values and can be regarded as global noises.

Two metrics are proposed to explore neuronal activity-related sources in an fNIRS dataset. To investigate neuronal activity-related source evoked by task stimuli, it often involves selecting SOI using a reference time course, which can be created by convolving the HRF and a square curve of task design of an experiment (McKeown et al., 1998). The Pearson correlation coefficient is used to compare the time course of sources and the generated reference time course. Task onsets and durations can be input to generate the reference time course when the user selects the metric of the reference time course. For RSFC detection, sources whose spatial map follows the hypothetic RSFC pattern and time course has prominent low-frequency (around the band of 0.01–0.1 Hz) spectrum are often selected (Cordes et al., 2001). The goodness of fit (GOF) metric is implemented to measure the similarity between the spatial map of the hypothesis and those of the separated sources:

(8) $$G\,O\,F\,\left(i\right)=\left|\frac{\text{y}\cdot {\text{c}}_{i}}{!\text{y}\cdot {\text{c}}_{i}}\right|,$$

in which **y** = [*y*_1_,*y*_2_,…,*y*_*n*_] denotes a vector of the Boolean variable representing the mask of the spatial template and ! is the logical operator of negation. In other words, users can let *y*_*i*_ = 1 if the *i*th channel belongs to the region of interest (ROI), otherwise *y*_*i*_ = 0. Spatial maps with high value inside ROI and low value outside ROI will have a high value of GOF.

#### Postprocessing

When using ICA to remove noises, the indices of noise sources are recorded, and the cleaned fNIRS data are reconstructed by discarding these noise sources using the equation:

(9) $$X_{c}=\sum_{j\in H}{\hat{\text{a}}}_{j}⊗{\hat{\text{s}}}_{j},$$

where *H*⊆{1,…,*m*} is the set including the indices of non-noise sources and *X_c_* is the noise-cleaned fNIRS data. The cleaned fNIRS data can then be analyzed by traditional methods such as GLM regression using other fNIRS software packages.

Using NIRS-ICA to explore SOI for a group of participants, the SOI is first selected for each participant based on methods mentioned in Section “Source Selection.” Then the group-level ICA results are derived using the selected source of each individual. Specifically, to view the group spatial map of the SOI, the individual spatial map is first transformed to z-map (Calhoun et al., 2001a). Then the group z-map is derived by averaging individual z-map, and the t-map is calculated by performing the two-tailed one-sample *t*-test (against zero) in a channel-wise manner (Zhang et al., 2010). The group-level time course is visualized based on the design of the experiment. For experiments that involve external task stimuli, the group-level time course of SOI is viewed by averaging individual time courses of SOI based on the task stimuli (Katura et al., 2008). Note that since the length of blocks may be different both within and across participants, the time courses are adjusted to have the minimum length of the blocks by removing the time points in the middle of the block. For the RSFC detection, since the sources of RSFC often have low-frequency spectrums, the group-level frequency spectrum of sources is shown instead of the time course (White et al., 2009).

### Validations

#### Simulative fNIRS Dataset for Noise Reduction

A simulative dataset is generated for validating the noise reduction mode of NIRS-ICA. Channels arranged as an 8 × 8 matrix are used for the detection of neuronal activity in an experiment with block-design tasks. Six sources, including one neuronal activity-related source and five noise sources, are generated for reconstructing the recorded fNIRS data using Eq. 1. Specifically, for the neuronal activity-related source, its temporal mode is simulated by convoluting a square wave of the task design (block-design) with HRF and its spatial mode is set to be locally distributed. For physiological noises, including heart rate, breath, Mayer wave, and low-frequency noise, their temporal modes are generated using sinusoidal waves with noise-specific frequencies (Supplementary Table 1). Their spatial modes are designed as global distribution. In addition, we simulate task-related motion artifacts on the edge of the channel matrix, which can lead to the detection of false-positive activation. Finally, channel-specific Gaussian noise is added to the data of each channel. Representative temporal and spatial modes of the simulated sources are shown in Supplementary Figure 1. The above simulations are repeated 10 times to produce a dataset acquired from a group of subjects. The detailed procedure of generating neuronal and noise sources can be found in the Supplementary Material. NIRS-ICA is used to remove noises for the generated dataset. The decomposition parameters of ICA are TICA and SOBI with 100 time-delayed correlation matrices. Ten sources are retained using PCA. To demonstrate noise reduction performance, both ICA-processed data and raw data are analyzed using the conventional GLM method implemented in NIRS-SPM (Ye et al., 2009). Preprocessing procedures, including a DCT-based detrending and HRF-based high-pass filtering implemented in NIRS-SPM, are used before GLM regression. Group-level β-map is calculated by averaging individuals’ β-maps and t-map is derived by performing a two-tailed one-sample *t*-test in a channel-wise manner.

#### Real fNIRS Dataset for Extracting Sources of Interest

We use previously conducted finger-tapping experiments to demonstrate the usages of extracting SOI (Zhao et al., 2020). Nine participants were recruited in the experiment and informed consent was obtained from all of them. The experiment consists of eight blocks of rest and task periods, each last 15 s. In the task period, participants performed a finger-tapping task, in which they alternatively pressed their index and ring finger on a keyboard. Eight sources and seven detectors were automatically arranged according to the transcranial brain atlas (TBA) to cover the pre- and post-central gyrus (see Supplementary Figure 2) (Zhao et al., 2020). LABNIRS (Shimadzu) system was used to record the brain activity of participants during the experiment. The recorded dataset is first preprocessed using DCT-based detrending and HRF-based high-pass filtering with NIRS-SPM. Then the preprocessed data are analyzed using conventional GLM regression and ICA implemented in NIRS-SPM and NIRS-ICA, respectively. The decomposition parameters of ICA are TICA and SOBI with 100 time-delayed correlation matrices. The number of sources is determined by retaining 99% of data variance using PCA (Zhang et al., 2010).

## Implementations and Results

### General Requirements and Main Interfaces

NIRS-ICA is developed using MATLAB 2012a (The MathWorks Inc., Natick, MA, United States), and had been tested in multiple platforms including Windows, MacOS, and Linux. To use NIRS-ICA, users need first install the MATLAB and add the NIRS-ICA folder to the search path, then tap “NIRS_ICA” in the MATLAB command line to open the main interface.

**FIGURE 2:**
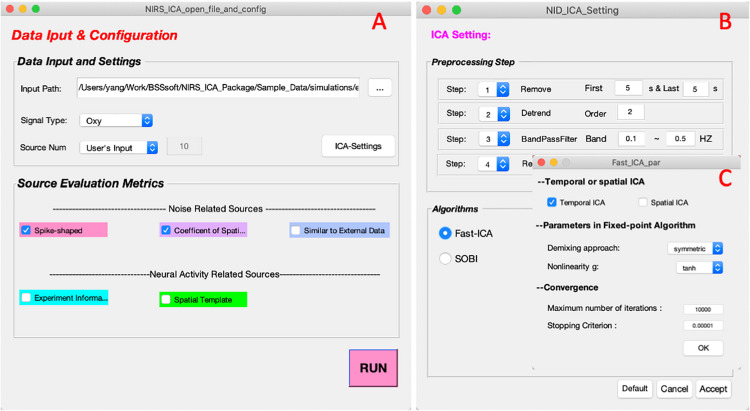
The interfaces of input and configuration of ICA processing. **(A)** The interface of data input and configuration. **(B)** The interfaces of ICA settings when users click the button of ICA-Settings in **(A)**. **(C)** The interface of setting parameters of FastICA.

The data input interface is implemented for inputting the fNIRS dataset and configuring the process of decomposition (Figure 2A). In the Data input and configuration panel, one can specify the input data, hemoglobin type to be processed, and the number of sources to be retained. The input data format supported by NIRS-ICA currently is the format converted using NIRS-KIT, which is a MATLAB toolbox for conducting conventional data analysis for fNIRS data developed by our group (Hou et al., 2021). NIRS-KIT provides a data conversion module for converting data recorded from various fNIRS devices. It also enables input probe montage information with the TopoMaker module, which is useful to visualize the spatial map of separated sources. The generation of probe montage used in the real fNIRS dataset (section “Real fNIRS Dataset for Extracting Sources of Interest”) is demonstrated in Supplementary Figure 3. If NIRS-ICA detects there is probe montage information in the input data structure, the spatial maps of the separated sources are displayed according to the probe montage. Otherwise, the spatial maps of the sources will be displayed as a matrix ordered by channel number.

Users can click the ICA settings button to specify the parameters of ICA processing (Figure 2B). NIRS-ICA provides commonly used preprocessing steps, including data crop, detrend, band-passed filtering, and subsampling, which can be selected before ICA. By clicking the aimed ICA algorithm in the Algorithm panel, one can select an ICA algorithm and set its associated parameters (Figure 2C). An example of parameters of preprocessing and the ICA algorithm is shown in Figure 2B. The Source evaluation metrics panel includes metrics for evaluating the separated sources based on the feature of their spatial maps or time courses. As discussed in Section “Source Selection,” three metrics for noise reduction and two metrics for selecting neuronal activity-related sources are implemented to evaluate the separated sources after ICA. Note that multiple evaluation metrics can be simultaneously selected.

**FIGURE 3:**
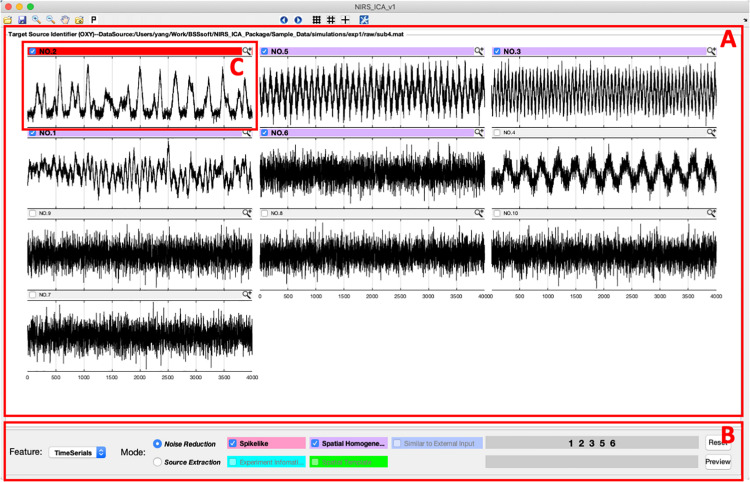
The interface of sources selection according to their time courses. **(A)** Sources displaying panel in which time courses of sources are depicted in a grid manner. **(B)** Displaying control panel for changing features, modes, or orders of displayed time courses. **(C)** A representative spike-shaped source is marked as a noise-related source.

**FIGURE 4:**
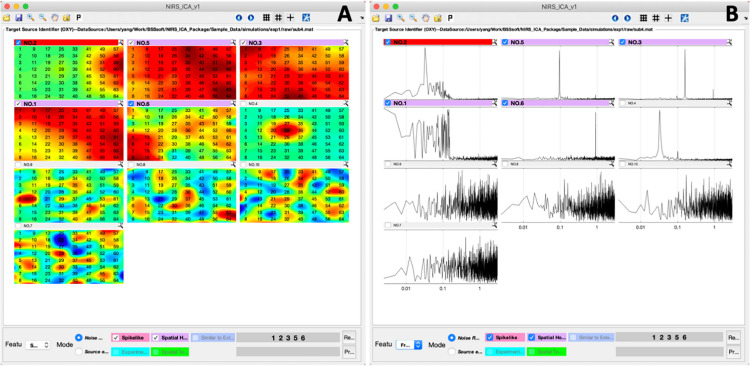
The interfaces of sources selection according to their spatial maps **(A)** and frequency spectrums of the time course **(B)**.

After input data and configuration for ICA processing, NIRS-ICA processes the input data using the selected preprocessing methods, decomposes the filtered data using the selected ICA algorithm and calculates the evaluation metrics of the separated sources, and opens the interface of source selection (Figure 3). User-friendly interfaces for selecting sources of noise or SOI are provided by NIRS-ICA. Users can switch between the mode of noise reduction and source extraction by clicking the radio button of Feature in the Display control panel (Figure 3B). The selection of the different modes of using NIRS-ICA will trigger different interfaces of detailed displaying the sources and results output, which will be demonstrated in later sections. The time courses of the separated sources are overviewed in a grid manner as shown in Figure 3. Users can choose to view source spatial maps or frequency spectrums of the time course by clicking the pop-up menu of the Features in the Displaying control panel (Figure 4). If evaluation metrics have been selected, separated sources are displayed according to the value of metrics in descending order; otherwise, they are shown by the output order of the selected ICA algorithm. If multiple metrics are chosen, the order is determined by averaging the normalized value of the selected metrics. Users can also choose whether to incorporate a metric by clicking its corresponding checkbox in the Display control panel. For each source, if the value of selected metrics surpasses a certain threshold, the top bar will be highlighted with a different color (instead of gray). Users can mark target sources by clicking the checkbox on the left of the top bar of each source. Sources can be displayed in detail by pressing the small magnifier on the right corner of the top bar. After the selection of sources, users can press the save icon in the menu bar to output the processed ICA results.

### Noise Reduction for the Simulative Dataset

A representative separation result of the simulative dataset is shown in Figure 3. It can be seen from the separation result that one source of spike-shaped and four sources of global noise are automatically labeled (top bars are highlighted in red and purple). Spike-shaped motion artifacts can be visually identified from the grid displaying the time courses (Figure 3C). From the spatial maps of sources, noises showing a global pattern (the sources with purple top bar) can be easily identified (Figure 4A). The time courses of the global noises also have narrow frequency bands as the generated true sources (Figure 4B).

**FIGURE 5:**
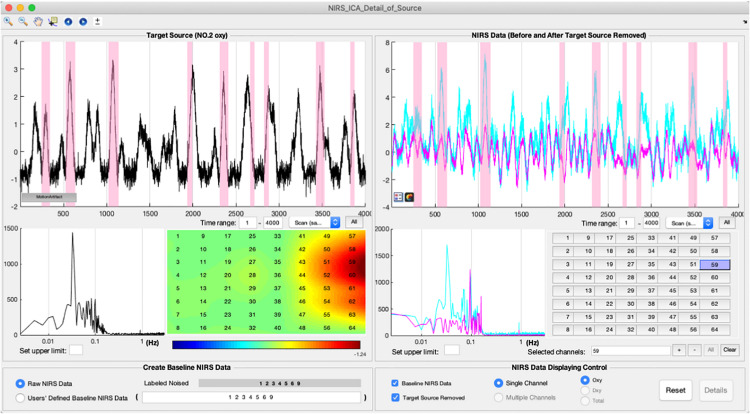
The interface of detailed displaying sources for noise reduction. A representative source of motion artifacts is depicted using the interface.

In the noise reduction mode, users can press the small magnifier on the top bar of the source to enter the interface of detailed displaying of a source (Figure 5). The detailed information of a separated source, including its time course, the frequency spectrum of the time course, and spatial map, is displayed in the left panel. To facilitate inspecting the relationship between the time course of the current source and the original signal of each fNIRS channel, the fNIRS time course is displayed in the right panel. Users can examine the contribution of the current source to the time course of each fNIRS channel by pressing the channel buttons on the right panel. To enable users to preview the performance of removing the current source, the cleaned fNIRS time course, i.e., reconstructed without the current source using Eq. 9, can be overlaid with the raw fNIRS time course by selecting the checkbox at the bottom of the left panel. The fNIRS time course or baseline time course displayed can also be the time course without the already-selected noise sources by selecting User-defined Baseline NIRS Data at the bottom of the left panel. The periods of motion artifact, i.e., MSD is larger than a threshold, in the time course of the source can be highlighted by pressing the button in the bottom left corner of the source time course. A representative source of motion artifact is shown in Figure 5. Its time course showed a spike-shaped waveform, and the spatial map has high values on the edge of the channel matrix, which are the same as the simulated true source. It can be seen that the spike-shaped waveform is greatly reduced after this source is removed in fNIRS data as shown in the right panel. After the selection of noise sources, users can press the preview button in the main interface to examine the data quality after the noise sources are removed (Figure 3B). Users are able to press the save button on the top menu of the main interface to save the cleaned fNIRS data, which is derived using Eq. 9. The saved fNIRS data has the same format as the input file, i.e., NIRS-KIT format, which facilitates further processing using other methods such as conventional GLM regression.

**FIGURE 6:**
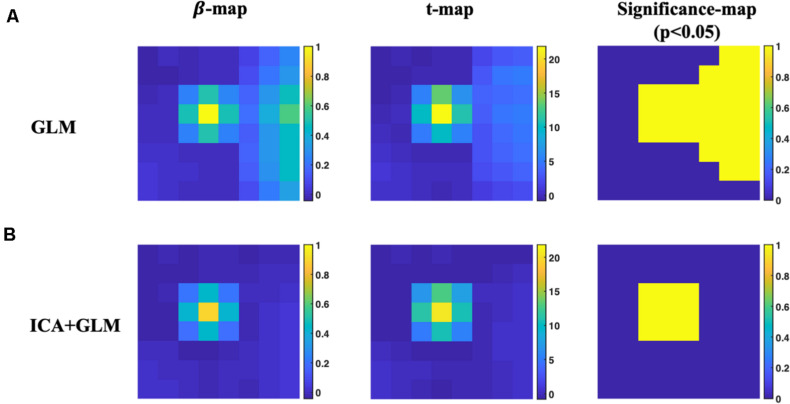
Group-level activation results of the simulated dataset. **(A)** The results of activation maps using conventional GLM analysis. **(B)** The results of activation maps using ICA preprocessing and conventional GLM analysis.

The group-level GLM results of both with and without ICA preprocessing are depicted in Figure 6. False-positive value can be found in β-map, T-map, and significance map (*p* < 0.05) due to the noise contamination using only conventional GLM analysis. With ICA preprocessing, the false-positive values are reduced and the resultant spatial maps of GLM are localized to the position of the simulated true sources.

### Extracting Neuronal Activity-Related Sources in the Real fNIRS Dataset

**FIGURE 7:**
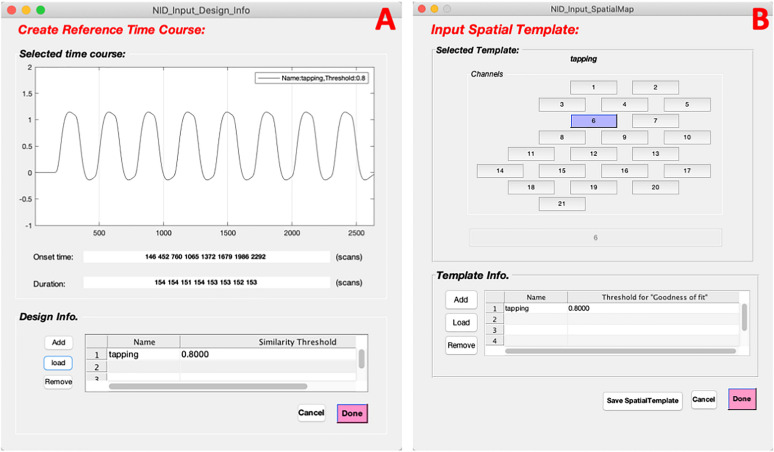
The interfaces of input reference time course and spatial template for extracting sources of interest (SOI). **(A)** The interface of creating a reference time course. **(B)** The interface of input a spatial template.

To facilitate extracting neuronal activity-related sources (SOI), NIRS-ICA provides interfaces to input reference time courses or spatial templates. Users can press the corresponding checkboxes in the Source evaluation metrics of Data input and configuration interfaces (Figure 2A), which enable creating reference time courses and spatial templates, respectively (Figure 7). By inputting or loading the onset times and durations of the task stimuli, the reference time course is generated as described in Section “Source Selection” (Figure 7A). The spatial template is created by selecting the channels within ROI in the user interface (Figure 7B). Users can generate multiple reference time courses or spatial templates since several task conditions or ROIs may be of interest in a study. The created reference time courses or spatial templates can also be saved as a MATLAB file and reloaded by pressing the load button on their respective interfaces.

**FIGURE 8:**
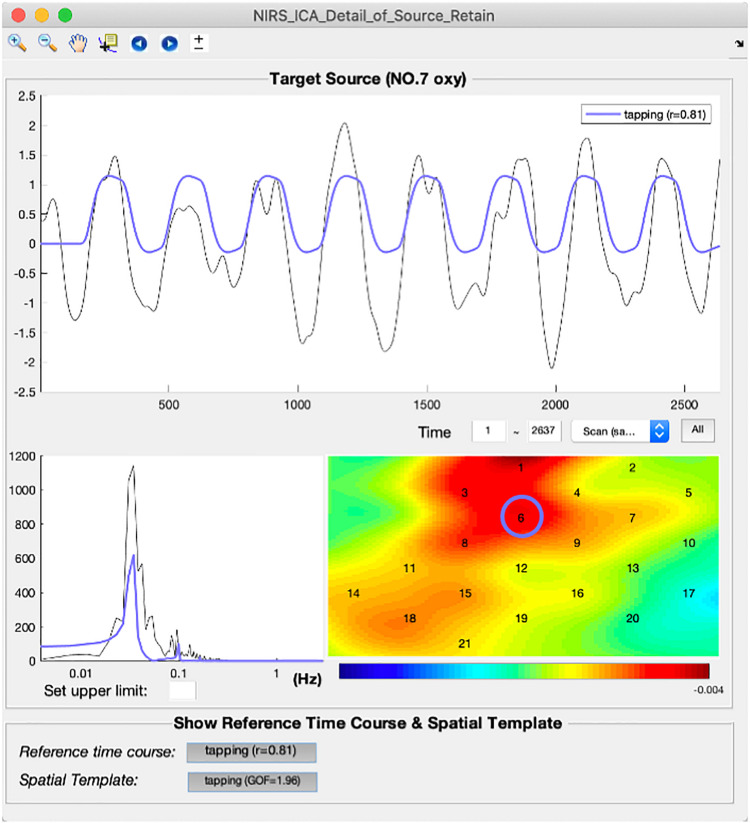
The interface of detailed displaying for extracting sources of interest.

A representative source of the task-evoked hemodynamic response of real fNIRS data is shown in the interface of the detailed displaying of SOI (Figure 8). By clicking the buttons on the bottom panel, the reference time course and spatial template are overlaid with the time course and spatial map of the current source, which enables users to examine the correlations between them in detail. It can be seen the time course of the representative source follows the reference time course consistently with a high correlation coefficient (*r* = 0.81). Its spatial map also depicts high value in channels within ROI (blue circle). After the selection of SOI for an individual participant, the selected source can be saved by pressing the save button on the top menu in the main interface (Figure 3).

**FIGURE 9:**
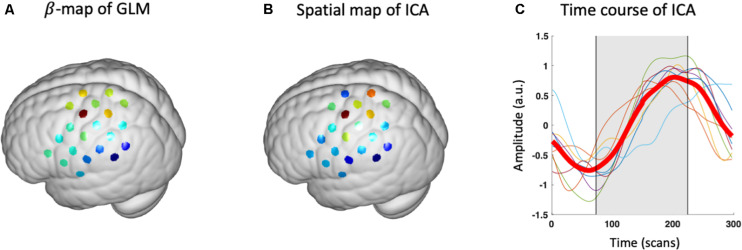
Group-level results of GLM and ICA. **(A) β** -map of conventional GLM analysis. **(B)** Group-level spatial map of ICA (z-map). **(C)** Group-level block-averaged time course (bold red line) and individual time courses (thin lines). Gray rectangle denotes the period of the task.

After the source of interest has been chosen for each participant, group-level SOI can be derived using the method described in Section “Postprocessing.” The averaged spatial map of SOI in brain space is visualized using a module inherited from NIRS-KIT (Figure 9). Note that users need to additionally input fiducial makers and channel locations recorded on a head model (or representative subject) using a 3D-digitizer, which is necessary to estimate brain locations measured by the channels (Hou et al., 2021). It can be seen the spatial map derived by ICA (z-map) is similar to the β-map of GLM, and both methods derive peak value at hand knob area, which is consistent with literature studies (Figures 9A,B). The block-averaged time course of ICA also depicts the hemodynamic response curve, which indicates that the current SOI is a task-evoked neuronal source (Figure 9C).

### Outputs

For both removing noise and extracting SOI, NIRS-ICA saves the parameters of the ICA process, the value of evaluation metrics, as well as the separation results in the output data structure. This output file can be reloaded to NIRS-ICA, which enables users to reproduce the ICA process and correct the source selection. The saved file can also be used to generate a document that includes the options used in the ICA processing. A representative document of the ICA processing used in analyzing the real fNIRS data is shown in Table 1. This document can be further reported in an fNIRS study that involves the ICA processing.

**TABLE 1:** Options of ICA processing generated by NIRS-ICA.

General information	Usage:	Extract sources of interest
	Hb type:	OXY
	Task or RSFC:	Task
	Number of participants:	9
ICA parameters	Number of sources:	PCA, 99%
	Algorithm:	SOBI
	Number of correlation matrices to be diagonalized:	100
Evaluation metrics	Similarity to the reference time course (R)	0.60 ± 0.16
	R for each participant	1(0.60), 2(0.40), 3(0.41), 4(0.66), 5(0.82), 6(0.70), 7(0.74), 8(0.67), 9(0.41)

## Discussion

In this study, we propose NIRS-ICA, a MATLAB toolbox for the applications of ICA for fNIRS studies. NIRS-ICA incorporates commonly used ICA algorithms for source separation, user-friendly GUIs, and quantitative evaluation metrics for source selection. It also supports two applications: noise reduction and exploration of neuronal activity-related sources in fNIRS signals. These functionalities are validated based on simulative and real fNIRS datasets. The GUIs and usages are also demonstrated based on the used datasets.

It should be noted that although ICA has been successfully applied in fNIRS studies, the interpretation of ICA results should be made with caution. ICA decomposes fNIRS data relying on the assumption of either temporal or spatial independence between sources of noise and sources of neuronal activity. Therefore, whether ideal decomposition can be achieved depends on how well these assumptions are satisfied in the fNIRS data to be processed. For example, fNIRS studies have shown that there is task-evoked physiological noise, which can temporally correlate with task-evoked hemodynamic response (Kirilina et al., 2012). In that case, TICA may fail to separate noise and neuronal activity-related sources. For SICA, since fNIRS often has a limited number of channels in the spatial dimension, how many channels are enough to obtain a reliable and reproducible decomposition needs to be investigated in future studies. The decomposition can be also influenced by the preprocessing parameters, the number of sources retained, and the separation algorithm used, which is still an open problem in the fNIRS field. Since the above factors will influence the ICA results, it is important to report these options for the reproducibility of an fNIRS study. With the assistant of NIRS-ICA, researchers can report the parameters for the decomposition, as well as the criterion for source selection, i.e., source evaluation metrics, thus improving the reproducibility of ICA applications for an fNIRS dataset, and further promoting the standardization of using ICA by the fNIRS community.

Several fNIRS toolboxes have already included ICA as a processing step of their analysis pipeline. For example, for data recorded by the NIRx system, one can use NICA and NAVI to denoise the fNIRS data or extracting features from the fNIRS data (Raggam, 2020). FastICA is also implemented in NyPin as a preprocessing step for noise reduction (Strangman et al., 2009). However, since these toolboxes are not dedicated to ICA processing, they have limitations such as availability for only certain types of devices, lack of decomposition algorithms, and methods facilitating source selection. Using the data conversion module provided by NIRS-KTI, NIRS-ICA supports various fNIRS devices such as ETG-4000/7000 (Hitachi Medical Company), LABNIRS (Shimadzu), or NIRx (Hou et al., 2021). It also includes two commonly used ICA algorithms and metrics facilitating source selection, which makes it more flexible and user-friendly than previously proposed toolboxes.

The future updates of NIRS-ICA will be focused on the following aspects: First, in the decomposition step, the current version of NIRS-ICA included two algorithms for source separation since they are commonly used and validated in fNIRS literature. Since the source separation performance may depend on the data quality, other algorithms such as infomax and JADE, which are widely used in other imaging modalities, may still be useful for fNIRS data (Correa et al., 2007). The decomposition performance can also be increased by adding external information, which can be acquired by additional devices. For example, multi-distance channels have been used to discriminate deep and shallow components in the ICA process (Funane et al., 2014). von Lühmann et al. (2019) also combined a 3D accelerometer with ICA to achieve a better noise reduction result. More preprocessing methods, such as excluding bad channels and data segmentation, will also be added. Second, in the source selection step, more evaluation metrics can be developed and included in the source selection step such as mean inter-block cross-correlation (MITC), which is used to select task-related sources (Katura et al., 2008). These metrics may potentially lead to automatic source selection, which will further reduce the subjectiveness in the source selection step. Third, since the current version of NIRS-ICA adopts manual source selection, the sources are manually matched between different subjects in the postprocessing step. Other automatic approaches of matching sources such as clustering and group-ICA will be included in the toolbox (Calhoun et al., 2001a; Spadone et al., 2012). Fourth, since ICA has been utilized in the domain of fNIRS hyper-scanning, it is hopeful to make NIRS-ICA supporting the analysis of multi-brain dataset so that it can be used for fNIRS hyper-scanning studies (Zhao et al., 2017). Finally, NIRS-ICA will soon be integrated into NIRS-KIT^1^, which is a toolbox for analyzing both task-related and resting-state fNIRS data using conventional methods developed by our group (Hou et al., 2021).

## Data Availability Statement

The data analyzed in this study are subject to the following licenses/restrictions: The data that support the findings of this study are available from the corresponding author upon reasonable request. Requests to access these datasets should be directed to C-ZZ, czzhu@bnu.edu.cn.

## Ethics Statement

The studies involving human participants were reviewed and approved by the State Key Laboratory of Cognitive Neuroscience and Learning, Beijing Normal University. The patients/participants provided their written informed consent to participate in this study.

## Author Contributions

YZ: conceptualization, methodology, data curation, formal analysis, and writing—original draft. P-PS: conceptualization, methodology, software, and resources. F-LT: methodology and resources. XH: software and resources. C-ZZ: conceptualization, methodology, supervision, and writing—review and editing. All authors contributed to the article and approved the submitted version.

## Conflict of Interest

The authors declare that the research was conducted in the absence of any commercial or financial relationships that could be construed as a potential conflict of interest.

## Footnotes

1 https://www.nitrc.org/projects/nirskit/
