# Supplementary material for: NIRS-ICA: A MATLAB Toolbox for Independent Component Analysis Applied in fNIRS Studies
Source: Front Neuroinform. 2021 Jul 14;15:683735. doi: 10.3389/fninf.2021.683735 (PMC8317505; doi:10.3389/fninf.2021.683735)
Supplement: Supplementary file 1 [file Data_Sheet_1.docx]

# Sources code and parameters of the ICA algorithms:

The parameters of decomposition algorithms implemented in NIRS-ICA are listed in Tables S1 and S2.

Table S1. Parameter of SOBI.

| Parameter | Description | Value (Default) |
| --- | --- | --- |
| Number of sample-delayed correlation matrices | Number of sample-delayed correlation matrices for joint diagonalization | 100 |

Source: Implementation from EEGLAB: <https://sccn.ucsd.edu/eeglab/download.php>

Table S2. Parameters of FastICA.

| Parameter | Description | Value (Default) |
| --- | --- | --- |
| TICA or SICA | Perform temporal ICA or spatial ICA | TICA |
| Demixing approach | Estimate components sequentially (defl) or in parallel (symm) | symm |
| g | The nonlinearity used in the objective function | tanh |
| Maximum number of iterations | The optimization stops when it does not converge in the maximum number of iterations | 10000 |
| Stop criterion | The algorithm converges when the difference of the value of objective function between two iterations is smaller than this value | 0.00001 |

Other parameters of FastICA are set as the default value, see the source code for more information.

Source: <http://www.cis.hut.fi/projects/ica/fastica>

# Generation of the simulative dataset:

The simulative dataset is generated to simulate a dataset recorded in a fNIRS experiment with block-designed task stimuli. The entire task period lasts 400s, which consists of 13 blocks of 15s rest and 15s task. 64 channels, arranged as an 8x8 square matrix, are used to sample to brain cortex with a sampling frequency of $fs=10\mathrm{Hz}$. Multiple time courses and spatial maps of sources are first generated and then they are used to reconstruct the fNIRS raw data using equation (1). After that channel-specific Gaussian noises (~$N(0,0.5)$) are added to the data of each channel. Representative sources are shown in Figure S1. Note that to control the amplitude of a source, the value of its spatial map is normalized using the maximum value of the spatial map, and the amplitude of its temporal mode is manipulated. The temporal and spatial modes of different sources are generated as follows:

Figure S1. Representative neuronal activity-related and noise sources in the simulative experiment.

**Source of neural-related hemodynamic response:**

The temporal mode of neural-related hemodynamic response is generated by convoluting a square wave of task stimuli (0 for rest, 1 for task) with hemodynamic response function (HRF), which is provided by NIRS-SPM (Ye et al., 2009). Spatial mode of this source is made by setting the element at row 4, column 5 of the 8x8 matrix to 1, and other elements to 0. Then, the matrix is smoothed by a 2×2 moving average spatial filter.

**Sources of physiological noise:**

The temporal mode of physiological noises is generated using sinusoidal waves with frequencies and amplitudes listed in Table S3. The phase of these sinusoidal is pseudo-randomized across different subjects. Note that for the Low-frequency physiological noise, its temporal mode is produced by combining sinusoidal waves with a frequency of 0 to 0.15Hz (step by 0.025) and pseudo-randomized phases. Spatial modes of the physiological noise are simulated using logical matrices with 1 assigned to 30 random channel positions. Then the matrices are smoothed by 5×5 moving average spatial filters.

**Source of motion artifacts:**

To generate task-related motion artifacts, we let the motion artifacts happened during the task period and influence the right edge of the channel matrix. Specifically, for the temporal mode, we first generate a vector of 0 with the same length as other sources and add 1 to the task period of the vector. The time points and number of artifacts (1) are pseudorandomized using uniform distributions, which are $t\sim U(t_{s},t_{e})$ and $n\sim U\left( 1,5 \right)$, where $t_{s}$ and $t_{e}$ are the starts and ends of the task stimuli. Then the vector is filtered using a Gaussian smoothing kernel with a standard deviation of 2s and scaled by 5. Spatial modes of motion artifacts are simulated by setting 1 to 3 pseudorandom positions ($\sim U(1,3)$) on the right edge of the channel matrix (other positions are 0), then the matrix is smoothed by a 5×5 moving average spatial filter.

Table S3. Frequency and amplitudes of the temporal mode of physiological noises.

| Noise type | Frequency (Hz) | Amplitudes (std) |
| --- | --- | --- |
| Low-frequency physiological noise | 0 - 0.15 | 1 |
| Mayer wave | 0.1 ± 0.02 | 0.5 |
| Breathing | 0.2 ± 0.03 | 0.5 |
| Heart rate | 1.1 ± 0.1 | 0.2 |

# Probe arrangement in the real fNIRS experiment:

Figure S2．Probe arrangement of the real fNIRS experiment. Optode and channel locations are depicted on a virtual scalp model (Ch2020) with pre- and post-central gyrus (AAL) provided by transcranial brain atlas (AAL).

The probe arrangement used in the real fNIRS experiment is shown in Figure S2. The channel locations in brain spaces of each individual subject were derived using 3D digitizer and individual structural MRI images. The channel positions depicted in Figure 9 are the mean positions of individual channel positions in the MNI space. One can see Zhao et. al. for detail of deriving channel locations in brain space (Zhao et al., 2020). When there is no individual scalp-brain correspondence information, users can use representative scalp brain correspondence to derive channel locations in brain space (Singh et al., 2005).

# Creating probe montage information using TopoMaker


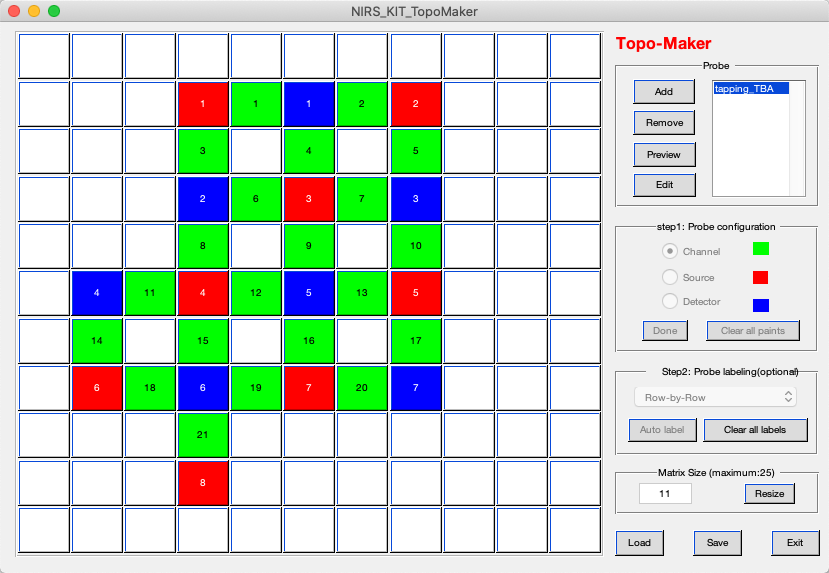


Figure S3. Adding probe montage information using the Topomaker module provided by NIRS_KIT.

The probe montage is generated according to the probe arrangement in the real fNIRS experiment (Figure S2).

# References:

Singh, A. K., Okamoto, M., Dan, H., Jurcak, V., and Dan, I. (2005). Spatial registration of multichannel multi-subject fNIRS data to MNI space without MRI. *NeuroImage* 27, 842–851. doi:10.1016/j.neuroimage.2005.05.019.

Ye, J. C., Tak, S., Jang, K. E., Jung, J., and Jang, J. (2009). NIRS-SPM: statistical parametric mapping for near-infrared spectroscopy. *NeuroImage* 44, 428–447. doi:10.1016/j.neuroimage.2008.08.036.

Zhao, Y., Xiao, X., Jiang, Y.-H., Sun, P.-P., Zhang, Z., Gong, Y.-L., et al. (2020). Transcranial brain atlas-based optimization for functional near-infrared spectroscopy optode arrangement: Theory, algorithm, and application. *Hum. Brain Mapp.* 2, 020801–1669. doi:10.1002/hbm.25318.
